# Progress in Understanding and Sequencing the Genome of *Brassica rapa*

**DOI:** 10.1155/2008/582837

**Published:** 2008-01-24

**Authors:** Chang Pyo Hong, Soo-Jin Kwon, Jung Sun Kim, Tae-Jin Yang, Beom-Seok Park, Yong Pyo Lim

**Affiliations:** ^1^Department of Horticulture, College of Agriculture and Life Science, Chungnam National University, Daejeon 305764, South Korea; ^2^Brassica Genomics Team, National Institute of Agricultural Biotechnology (NIAB), Rural Development Administration (RDA), Suwon 441707, South Korea; ^3^Department of Plant Science, College of Agriculture and Life Sciences, Seoul National University, Seoul 151921, South Korea

## Abstract

*Brassica rapa*, which is closely related to *Arabidopsis thaliana*, is an important crop and a model plant for studying genome evolution via polyploidization. We report the current understanding of the genome structure of *B. rapa* and efforts for the whole-genome sequencing of the species. The tribe *Brassicaceae*, which comprises ca. 240 species, descended from a common hexaploid ancestor with a basic genome similar to that of *Arabidopsis*. Chromosome rearrangements, including fusions and/or fissions, resulted in the present-day “diploid” *Brassica* species with variation in chromosome number and phenotype. Triplicated genomic segments of *B. rapa* are collinear to those of *A. thaliana* with InDels. The genome triplication has led to an approximately 1.7-fold increase in the *B. rapa* gene number compared to that of *A. thaliana*. Repetitive DNA of *B. rapa* has also been extensively amplified and has diverged from that of *A. thaliana*. For its whole-genome sequencing, the *Brassica rapa* Genome Sequencing Project (*Br*GSP) consortium has developed suitable genomic resources and constructed genetic and physical maps. Ten chromosomes of *B. rapa* are being allocated to *Br*GSP consortium participants, and each chromosome will be sequenced by a BAC-by-BAC approach. Genome sequencing of *B. rapa* will offer a new perspective for plant biology and evolution in the context of polyploidization.

## 1. IMPORTANCE OF *BRASSICA* GENOMICS

The genus *Brassica* is one of the core genera in the tribe *Brassicaceae* and includes a number of crops with wide adaptation under a variety of agroclimatic conditions. Economically, *Brassica* species are important sources of vegetable oil, fresh, preserved vegetables, and condiments. *B. napus, B. rapa*, *B. juncea,* and *B. carinata* provide about 12% of the worldwide edible vegetable oil supply [1]. The *B. rapa* and *B. oleracea* subspecies represent many of the vegetables in our daily diet. In particular, *B. rapa* ssp. *pekinensis* (Chinese cabbage), on which this article focuses, is one of the most widely used vegetable crops in northeast Asia. Moreover, *Brassica* species are important sources of dietary fiber, vitamin C, and anticancer compounds [2].

**Figure 1:**
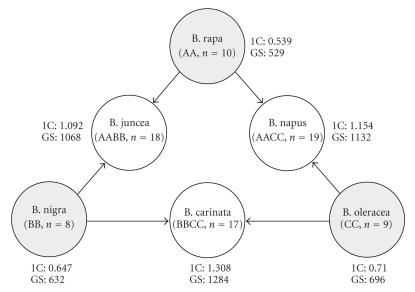
Genetic relationship of the different diploid and amphidiploid *Brassica* species. 1C, 1C nuclear DNA content (pg); GS, genome size (Mbp) [3, 4].

The genetic relationships among the different diploid and amphidiploid *Brassica* species are described by the U’s triangle [3]. Of the six widely cultivated species of *Brassica*, *B. rapa* (AA, 2*n* = 20), *B. nigra* (BB, 2*n* = 16), and *B. oleracea* (CC, 2*n* = 18) are monogenomic diploids. The remaining three species, *B. juncea* (AABB, 2*n* = 36), *B. napus* (AACC, 2*n* = 38), and *B. carinata* (BBCC, 2*n* = 34) exhibit stable diploid genetics, but are allotetraploids, which have evolved via hybridization between differing monogenomic diploids [3]. The diploid *Brassica* genomes range from 1.1 pg/2C (529 Mbp/1C) for *B. rapa* to 1.4 pg/2C (696 Mbp/1C) for *B. oleracea* (see Figure 1) [4]. The genomes of the allotetraploids range from 2.2 pg/2C (1,068 Mbp/1C) for *B. juncea* to 2.6 pg/2C (1,284 Mbp/1C) for *B. carinata* (see Figure 1).

The genus *Brassica* is characterized by morphological diversity with regard to inflorescences, leaves, stems, roots, and terminal or apical buds [5]. For example, such morphological diversity can be easily observed in subspecies of *B. oleracea*: the enlarged inflorescences of cauliflower (*B. oleracea* ssp. *botrytis*) and broccoli (*B. oleracea* ssp. *italica*); the enlarged stems of kohlrabi (*B. oleracea* ssp. *gongylodes*) and marrowstem kale (*B. oleracea* ssp. *medullosa*); and the many axillary buds of Brussels sprout (*B. oleracea* ssp. *gemmifera*) [5]. The morphological diversity in *Brassica* species may be linked to genomic changes associated with polyploidization [6]. The polyploidization in *Brassica* species has brought about triplication of genomic segments and subsequent rearrangements such as inversions, insertions, deletions, and substitutions [7–16], and these genetic variations may cause novel phenotypic variations for traits among these species [5, 6]. Thus, *Brassica* genomics will provide us with an understanding of the rapid phenotypic evolution of polyploid plants. Additionally, it will help us to understand genomic changes and how they shape the allotetrapolyploid *Brassica* species. For example, a study has been done looking at rapid genomic changes and the effect of nuclear-cytoplasm interaction in synthetic allotetrapolyploid species [17].

Because of the high economic value of *Brassica* species throughout the world and their potential to be models for the study of polyploidization, genome sequencing projects for *Brassica* species, especially *B. rapa* and *B. oleracea*, have recently been initiated (http://www.brassica.info) [18–20]. In particular, *B. rapa* ssp. *pekinensis* inbred line Chiffu-401-42, discussed in this article, has been selected for *Brassica*-A genome sequencing in the *Brassica* *rapa* Genome Sequencing Project (*Br*GSP) (http://www.brassica.info), a component of the consortium of the Multinational *Brassica* Genome Project, with the goal of completely sequencing this genome through a BAC-by-BAC approach. The *Br*GSP consortium has developed genomic resources for this purpose and is proceeding with whole-genome sequencing.

## 2. CURRENT UNDERSTANDING OF THE GENOME STRUCTURE OF *B. RAPA*

### 2.1. Karyotype of *B. rapa*

Karyotyping is the starting point for understanding the genome structure of a species. Moreover, it provides insight into genome evolution. Most of the karyotypic analyses in *B. rapa* have been performed on mitotic metaphase chromosomes [21–24]. However, the analyses are limited in what they can reveal about the cytological structure of the genome because of the low resolution of the technique. For example, different measurements of chromosome lengths and rDNA loci are obtained by this method. Recently, the high-resolution karyotype for the *B. rapa* ssp. *pekinensis* inbred line Chiffu was determined on pachytene chromosomes by using 4′-6-diamino-2-phenylindole dihydrochloride (DAPI) staining and fluorescence in situ hybridization (FISH) of rDNAs and pericentromeric satellite repeats [25]. By DAPI analysis, the mean lengths of ten pachytene chromosomes ranged from 23.7 *μ*m to 51.3 *μ*m, with a total of 385.3 *μ*m, a total length which is 11.9- ∼17.5-fold longer than that of the mitotic metaphase chromosomes reported by Lim et al. [24] and Koo et al. [25]. In comparison, pachytene chromosome length of *A. thaliana*, *Medicago truncatula*, and tomato was estimated to be about 7.4%, 15%, and 24% of the total pachytene chromosome length, respectively (reviewed in Koo et al. [25]). In *B. rapa*, the pachytene karyotype consists of two metacentric (chromosomes 1 and 6), five submetacentric (chromosomes 3, 4, 5, 9, and 10), two subtelomeric (chromosomes 7 and 8), and one acrocentric chromosome (chromosome 2), with the corresponding centromeric index ranges of 38.8–41.0%, 29.5–36.7%, 17.4–20.2%, and 9.38%, respectively [25]. In the chromosomal structure at pachytene, the total length of pericentromeric heterochromatin regions was estimated to be 38.2 *μ*m, which is approximately 10% of the total chromosome length [25]. In conjunction with chromosomal structure and characteristics, 5S rDNA loci were located on pericentromeric regions of the short arms of chromosomes 2 and 7 as well as the long arm of chromosome 10, while 45S rDNA loci were located on the short arms of chromosomes 1, 2, 4, and 5 as well as the long arm of chromosome 7 [24, 25].

### 2.2. Collinearity between genomic segments of *Arabidopsis* and *Brassica*

**Figure 2:**
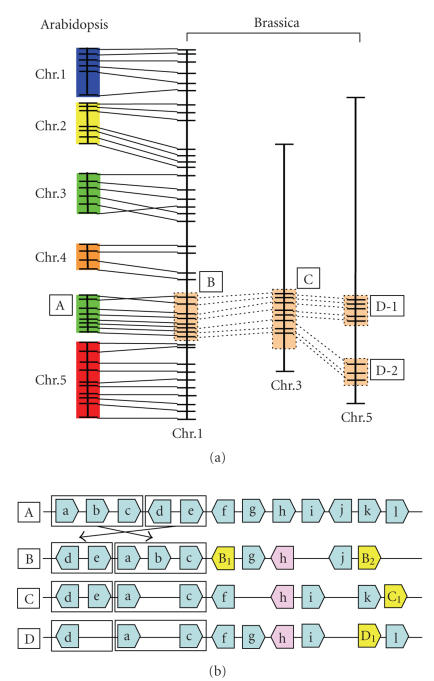
An example of a comparative map of *Arabidopsis* and *Brassica*. (a) Collinearity between genomic segments of the two species and genome triplication of *Brassica* revealed by comparative genetic mapping. (b) Synteny of genes in a triplicated genomic region of *Brassica*.

*Brassica* species are closely related to *A. thaliana*, having diverged 17-18 million years ago (MYA) from their common ancestor [16]. *A. thaliana*, which has been completely sequenced, has a rather small genome (about 146 Mb) with relatively little repetitive DNA and a high gene density [26, 27]. Protein-coding regions of the genomes of *Brassica* species show high sequence conservation with those of *A. thaliana*, with nucleotide sequence similarity in exons between *B. oleracea* and *A. thaliana* ranging from 75% to 90%, compared to *<*70% for intronic regions [28]. This similarity allows the identification of sets of candidate genes in *Brassica* species and the studying of their genome structures through comparative genomics [29]. Comparative studies between *Arabidopsis* and *Brassica* have revealed the presence of collinear chromosome segments (see Figure 2). Comparative genetic mapping between diploid *Brassica* species and *A. thaliana* to identify homologous loci have revealed many conserved blocks in their genomes [7, 8, 14, 30]. Comparative physical mapping between *Arabidopsis* and *Brassica* further corroborated the findings. A set of six bacterial artificial chromosomes (BACs), representing a 431-kb contiguous region of *Arabidopsis* chromosome 2, was mapped on chromosomes and DNA fibers of *B. rapa* [31]. Moreover, studies on a 222-kb gene-rich region of *A. thaliana* chromosome 4 and its homologous counterparts in *B. rapa* or *B. oleracea* revealed the collinearity of genes in homologous segments [9, 11, 13]. This finding was supported by sequence analysis of specific homologous genomic segments [15, 16]. However, many structural rearrangements differentiate the *Brassica* and *Arabidopsis* chromosomes (see Figure 2). Comparative genetic mapping between *B. nigra* and *A. thaliana* species revealed that the average length of conserved segments between the two species was estimated at about 8 cM, which corresponds to ∼90 rearrangements since the divergence of the two species [7]. In addition, it was found that gene contents in their homologous genomic segments were also variable with interstitial gene losses and insertions [9, 11, 13, 15, 16].

### 2.3. Genome triplication of diploid *Brassica* species

Most of the comparative studies mentioned above demonstrated that *Brassica* species contain extensively triplicated counterparts of the corresponding homologous segments of the *A. thaliana* genome (see Figure 2), thereby suggesting that diploid *Brassica* species may have been derived from a hexaploid ancestor: the genome which was similar to *Arabidopsis*. Consistent with the nature of genome triplication, Yang et al. [16] reported that paralogous subgenomes of diploid *Brassica* species triplicated 13∼17 MYA, very soon after the *Arabidopsis* and *Brassica* divergence that occurred at 17∼18 MYA. In addition, it was reported that after the *Brassica* genomes had triplicated, their subgenomes were rearranged by inversions, translocations [7, 12, 32], extensive interspersed gene loss, as well as gene insertions occurred relative to the inferred structure of the ancestral genome (see Figure 2). Additionally, such genome triplication was extensively found across the tribe *Brassicaceae* [12]. In comparison with the genome of *A. thaliana*, the genome triplication in *Brassica* species has clearly led to an increase in the genome size, resulting in a 3- to 5-fold inflation.

Genome triplication events in *Brassica* species may also have an effect on gene expression of multicopy genes, leading to such phenomena as pseudogenization, subfunctionalization, or neofunctionalization in species [33–38]. For example, the MADS-box transcription factor family, whose members control key aspects of plant vegetative and reproductive development, shapes genetic systems by subfunctionalization [37]. It appears that after polyploid formation, considerable and sometimes very rapid changes in genome structure and gene expression have occurred. Researchers have hypothesized that genomic triplication in *Brassica* species permits mutations in loci that are normally under tight selective constraints in *Arabidopsis*, and may thus result in the observed greater phenotypic plasticity in *Brassica* [5]. Studies on expression of duplicated genes in *Brassica* species will provide insight into the role of polyploidization in the phenotypic divergence of the plant genus.

### 2.4. Survey of the *B. rapa* genome revealed by BAC-end sequence analysis

The *B. rapa* genome was surveyed via the analysis of its 12,017 *Hin*dIII BAC-end sequences (Table 1) [39]. Analyses of BAC-end sequence or genome survey sequences assist in understanding whole genome structure [39–41]. It was estimated that the *B. rapa* genome might contain about 43000 genes (covering 16.8% of the genome), 1.6 times more than the *A. thaliana* genome. Recently, Yang et al. [16] also estimated the gene content of *B. rapa* to range from 49,000 to 63,000, based on predictions from microsynteny studies. It has been suggested that chromosomal triplication events in *Brassica* have led to an increase in gene number with subsequent gene loss [15, 16, 39, 42].

Transposable elements (TEs) with a predominance of retrotransposons were estimated to occupy approximately 14% of the genome (covering approximately 74 Mb), 8.2 times greater than that observed previously in *A. thaliana* [44]. Zhang and Wessler [44] reported that TEs in *B. oleracea* constituted 20% of the genome, slightly more than what was predicted for the *B. rapa* genome. Of the predicted TEs, LTR retrotransposon families were the most abundant (69.9%), followed by non-LTR retrotransposons (13.4%), DNA transposons (11.4%), and other retrotransposons (5.3%). In particular, *Ty1*/*copia*-like and *Ty3*/*gypsy*-like retrotransposons occupied 39.5% and 30.2% of LTR retrotransposon families, respectively. The amplification of TEs in *B. rapa*, especially retrotransposons, may have played a crucial role in both evolution and genomic expansion.

Simple sequence repeats (SSRs) have been estimated to occur with a frequency of approximately one per 4.8 kb within the *B. rapa* genome, as compared to approximately one per 3.2 kb within the *A. thaliana* genome [39]. Of SSRs identified, trinucleotides were the most abundant repeat type, constituting about 37% of all SSRs, a percentage similar to those reported in other plant genomes [39, 45]. Comparison of SSR densities in different genomic regions demonstrated that SSR density was greatest immediately in 5′-flanking regions of predicted genes [45]. SSRs were also preferentially associated with gene-rich regions, with pericentromeric heterochromatin SSRs mostly associated with retrotransposons [45], suggesting that the distribution of SSRs in the genome is nonrandom [39, 45].

### 2.5. Structure of (peri)centromeres of *B. rapa*

The centromere is a dynamic and rapidly evolving structure and consists largely of highly repetitive DNA sequences, especially tandem satellite repeats and retrotransposons [46, 47]. Centromeric repeats characterized in plant genomes are composed of 155∼180-bp tandem repeat motifs, including the 180-bp pAL1 satellite in *A. thaliana* [48–50], the 155∼165-bp CentO satellite in rice [51, 52], the 156-bp CentC satellite in maize [53] and the 169-bp satellite in *Medicago truncatula* [54, 55]. Centromeric satellite repeats of *Brassica* species, except for those of *B. nigra*, are represented by the 176-bp CentBr [24, 25, 56–59]. The CentBr repeats in the *B. rapa* genome belong to two classes which have 82% sequence similarity. The two classes are chromosome-specific, with CentBr1 found on eight chromosomes (chromosomes 1, 3, and 5–10) and CentBr2 on two chromosomes (chromosomes 2 and 4) [24, 25, 39]. Such distribution of the CentBr family may reflect the predominance of CentBr1 in the *Brassica* genome [39]. The CentBr repeats have also undergone rapid evolution within the *B. rapa* genome and have diverged among the related species of *Brassicaceae* [39]. Recently, Lim et al. [59] identified and characterized the major repeats in centromeric and pericentromeric heterochromatin of *B. rapa*. The region contains CentBr arrays, 238-bp degenerate tandem repeat (TR238) arrays, rDNAs, centromere-specific retrotransposons of *Brassica* (CRB), and pericentromere-specific retrotransposons (PCRBr). In particular, CRB was a major component of all centromeres in three diploid *Brassica* species and their allotetraploid relatives, and PCRBr and TR238 were A-genome-specific [59].

## 3. PROGRESS OF *B. RAPA* GENOME SEQUENCING

### 3.1. Genomic resources

The development of genomic resources is a prerequisite to undertaking genome sequencing in any crop species. Genomic resources, including reference mapping populations, DNA libraries, and DNA sequences have been developed for *B. rapa* ssp. *pekinensis* inbred line Chiffu-401-42 (Table 2). Two reference mapping populations were derived from two *B. rapa* ssp. *pekinensis* inbred lines, Chiifu-401-42 and Kenshin-402-43 (CK), and comprise 78 double haploid (DH) lines (the CKDH population) and 201 recombinant inbred (RI) lines (the CKRI population). These mapping populations have been used for construction of reference genetic maps for genome sequencing [20]. The bacterial artificial chromosome (BAC) system, commonly used for developing large-insert DNA libraries, is an invaluable resource for structural and functional genomics. Three Chiffu BAC libraries were constructed by using restriction enzymes: *Hin*dIII, *Bam*HI, and *Sau*3AI, and designated as KBrH, KBrB, and KBrS. These libraries consist of 56592, 50688, and 55296 clones with an average insert size of 115 kb, 124 kb, and 100 kb, respectively. These BAC libraries cover approximately 36 genome equivalents, assuming that the genome size of Chinese cabbage is 529 Mb. Using these BAC clones, the *Br*GSP community has recently generated a total of 200017 BAC-end sequences. In combination with BAC fingerprinting data, the BAC-end sequences will give insight into the structure of the genome, be a resource for development of genetic markers, and aid in finding the BAC clones that correspond to the minimal tilling paths in genome sequencing [19, 60, 61]. For functional genomics of *B. rapa*, 22 cDNA libraries from different tissues, including leaves, roots, cotyledons, stems, seedlings, ovules, siliques, and anthers of Chiffu, have been constructed, and a total of 128582 expressed sequence tags (ESTs) have been generated from these cDNA libraries (GenBank accession number CO749247∼CO750684 and EX015357∼EX142500). Currently, the ESTs have been used for construction of *B. rapa* unigene set and gene expression microarray (http://www.brassica-rapa.org).

### 3.2. Genetic and physical mapping

**Figure 3:**
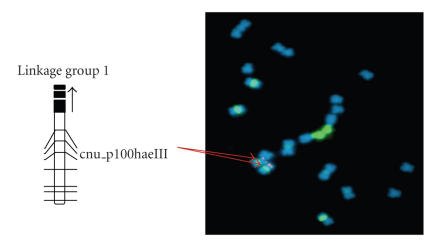
An example of an alignment of linkage group 1 in the reference genetic map to the corresponding chromosome 5 through FISH using locus-specific BAC clones.

Some genetic linkage maps of *B. rapa*, on which genetic markers were distributed over ten linkage groups, have been constructed since 1990 [62–67] (summarized in Table 3). The distances of genetic linkage maps ranged from 890 cM to 1850 cM. However, the genetic linkage maps may not provide direct and accurate genetic information for the Chiffu genome sequencing because of genetic variation between the mapping populations. For that reason, the *Br*GSP community has constructed the CK genetic linkage map. Using the 78 CKDH lines, a reference genetic linkage map has been constructed [67]. The map consists of a total of 556 markers, including 278 AFLPs, 235 SSRs, 25 RAPDs, and 18 ESTPs/STS/CAPS markers. Ten linkage groups were identified and designated as R1 to R10 via mapping with SSR markers derived from the reference linkage map of *B. napus* reported previously [68] (Table 4). The total length of the linkage map was 1182 cM with an average interval of 2.83 cM between adjacent loci. Recently, for high-resolution genetic mapping, the community has set a goal of developing more than 1,000 SSR markers derived from BAC-end sequences, ESTs, and BACs. Moreover, based on the sequence-tagged site (STS) markers, a CKRI genetic linkage map has been constructed to be complementary to the CKDH one. The linkage groups in these genetic maps may not correspond to the chromosomes assigned in the cytogenetic map. Therefore, it is important to align the linkage groups on the genetic map with chromosomes of the cytogenetic map. All ten linkage groups of a reference genetic map of *B. rapa* are being assigned to the corresponding chromosomes through fluorescence in situ hybridization (FISH) using locus-specific BAC clones as probes (see an example in Figure 3, unpublished data).

The fingerprinted BAC map (so-called “physical map”) makes it possible to select clones for sequencing that would ensure comprehensive coverage of the genome and reduce sequencing redundancy [69]. In addition, the clone-based map also enables the identification of large segments of the genome that are repeated, thereby simplifying the sequence assembly. To construct a deep-coverage BAC physical map of the *B. rapa* genome, all BAC clones from the three BAC libraries were fingerprinted using restriction enzyme digestion and SNaPshot [70] methodologies, and then BAC contigs have been assembled by FingerPrinted Contigs (FPC) software (http://www.agcol.arizona.edu/software/fpc/). This data will be open to the *Brassica* *rapa* genome sequencing consortium.

### 3.3. Approach to genome sequencing

Seed BACs for genome sequencing have been selected through in silico allocation of *B. rapa* BAC-end sequences onto counterpart locations of *Arabidopsis* chromosomes [19]. Of 91000 BAC-end sequences, a total of 45232 showed significant sequence similarity with unique *Arabidopsis* sequences, and 4317 BAC clones were allocated on *Arabidopsis* chromosomes by significant matching with both ends within 30–500 kb intervals, which span 93 Mb of *Arabidopsis* euchromatin regions (covering 78.2% of the *Arabidopsis* genome). However, approximately 9.4 Mb of euchromatin regions and 16.6 Mb pericentromeric heterochromatin regions of the *Arabidopsis* genome were not covered by the *B. rapa* BAC span (span is considered by best hit of paired ends). Based on the physical map of *B. rapa* and the in silico comparative map of its BAC-ends onto *Arabidopsis* chromosomes, 629 seed BACs have been selected spanning 86 Mb of *Arabidopsis* euchromatin regions and scattered throughout the *B. rapa* genome (http://www.brassica-rapa.org), and the BACs have been mapped on *B. rapa* chromosomes by STS mapping and FISH analysis. The seed BACs which are anchored and sequenced will be used as stepping stones for sequencing of the ten chromosomes.

Considering the large genome size and the possibility of international cooperation, a chromosome-based approach was suggested. Of ten chromosomes (or linkage groups), eight have been allocated to the participating countries as follow: Korea (R3 and R9), Canada (R2 and R10), UK and China (R1 and R8), USA (R6), and Australia (R7). However, R4 and R5 have remained unassigned. Progress of chromosome sequencing will be reported soon by each country.

## 4. CONCLUSIONS

*Brassica* species are economically important crops and serve as model plants for studying phenotypic evolution associated with polyploidization. The *Brassica* genomes have extensively triplicated and undergone subsequent genome rearrangements with sequence variations. This has significantly affected their genome structure and may underline phenotypic diversity. Genome sequencing of *B. rapa* can pave the way for elucidation of the relationship between genome evolution and phenotypic diversity. Moreover, it enables us to search for genes and develop molecular markers associated with agricultural traits, thereby establishing a molecular breeding system contributing to improvement of *Brassica* species economically.

## Figures and Tables

**Table 1:** Comparison of gene, TE, and SSR abundances in *B. rapa* and *A. thaliana*.

Contents	*B. rapa*	*A. thaliana*	References
Genome size (Mb)	529	146	[4, 27]
Gene number	4300∼63000	26,207	[16, 39, 43]
TE abundance (%)^(1)^	13.8	6∼7	[39, 44]
SSR number	≈110,000 (one SSR/4.8 kb)	≈36,756 (one SSR/3.2 kb)	[39, 45]

^(1)^Coverage of TEs in the genome.

**Table 2:** Genomic resources for whole-genome sequencing of *B. rapa*.

Genomic resources	Source material	Number
Mapping populations		
* *DH line	Chiffu-401-42 × Kenshin-402-43	78 lines (F_2_ generation)
* *RI line	Chiffu-401-42 × Kenshin-402-43	201 lines (F_8_ generation)
BAC libraries		
* * *Hin*dIII (KBrH)	Chiffu-401-42	56592 clones (115 kb^(1)^)
* * *Bam*HI (KBrB)	Chiffu-401-42	50688 clones (124 kb^(1)^)
* * *Sau*3AI (KBrS)	Chiffu-401-42	55296 clones (100 kb^(1)^)
cDNA libraries		
* *22 cDNA libraries	Different tissues of Chiffu-401-42 and Jangwon including leaves, roots, cotyledons, stems, seedlings, ovules, siliques, anthers	—
BAC-end sequences	KBrH, KBrB, and KBrS clones	200017 sequences
ESTs	22 cDNA clones	129928 sequences
BAC shotgun sequences	KBrH, KBrB, and KBrS clones	on-going^(2)^

^(1)^Average insert size (kb). ^(2)^Of BACs sequenced, 511 BACs have been deposited in GenBank.

**Table 3:** Genetic linkage maps of *B. rapa* developed since 1990.

Mapping population	Population type	Population size	No. of loci	Type of markers	Total length of map (average interval)	References
Michihili × Spring broccoli	F_2_	95	280	RFLP	1850 cM (6.6 cM)	[62]
Per (winter turnip rape)× R500 (spring yellow sarson)	F_2_	91	139	RFLP	1785 cM (13.5 cM)	[63]

Per (winter turnip rape)× R500 (spring yellow sarson)	F_6_RI	87	144	RFLP	890 cM (6.0 cM)	[64]

Developed from Chinese cabbage F_1_ cultivar Jangwon	F_2_	134	545	RFLP, SSR	1287 cM (2.4 cM)	[65]

G004 (CR^(a)^ DH line)× A9709 (CS^(b)^ DH line)(cultivars of Chinese cabbage)	F_2_	94	262	RFLP, SSR, RAPD	1005 cM (3.7 cM)	[66]

Chiffu-401-42× Kenshin-402-43	DH	78	556	AFLP, SSR, RADP, ESTP, STS, CAPS	1182 cM (2.83 cM)	[67]

**Table 4:** The correspondence between genetic linkage groups of *B. rapa* ssp. *pekinensis* based on *B. napus* reference linkage maps.

Genetic linkage map of *B. napus* [68]	Choi et al. [67]	Kim et al. [65]	Suwabe et al. [66]
A1 (N1)	R1	R1	LG6
A2 (N2)	R2	R2	LG8
A3 (N3)	R3	R3	LG1
A4 (N4)	R4	R4	LG10
A5 (N5)	R5	R5	LG3
A6 (N6)	R6	R6	LG2
A7 (N7)	R7	R7	LG4
A8 (N8)	R8	R8	LG7
A9 (N9)	R9	R9	LG5
A10 (N10)	R10	R10	LG9
